# Benznidazole Anti-Inflammatory Effects in Murine Cardiomyocytes and Macrophages Are Mediated by Class I PI3Kδ

**DOI:** 10.3389/fimmu.2021.782891

**Published:** 2021-12-02

**Authors:** Ágata C. Cevey, Paula D. Mascolo, Federico N. Penas, Azul V. Pieralisi, Aldana S. Sequeyra, Gerardo A. Mirkin, Nora B. Goren

**Affiliations:** ^1^ Universidad de Buenos Aires, Facultad de Medicina, Departamento de Microbiología, Parasitología e Inmunología, Buenos Aires, Argentina; ^2^ CONICET, Universidad de Buenos Aires, Instituto de Investigaciones Biomédicas en Retrovirus y SIDA (INBIRS), Buenos Aires, Argentina; ^3^ CONICET, Universidad de Buenos Aires, Instituto de Investigaciones en Microbiología y Parasitología Médica (IMPaM), Buenos Aires, Argentina

**Keywords:** benznidazole, anti-inflammatory effects, PI3K – AKT pathway, macrophages (M1/M2), inflammatory mediators, cardiomyocytes

## Abstract

Benznidazole (Bzl), the drug of choice in many countries for the treatment of Chagas disease, leads to parasite clearance in the early stages of infection and contributes to immunomodulation. In addition to its parasiticidal effect, Bzl inhibits the NF-κB pathway. In this regard, we have previously described that this occurs through IL-10/STAT3/SOCS3 pathway. PI3K pathway is involved in the regulation of the immune system by inhibiting NF-κB pathway through STAT3. In this work, the participation of PI3K in the immunomodulatory effects of Bzl in cardiac and immune cells, the main targets of Chagas disease, was further studied. For that, we use a murine primary cardiomyocyte culture and a monocyte/macrophage cell line (RAW 264.7), stimulated with LPS in presence of LY294002, an inhibitor of PI3K. Under these conditions, Bzl could neither increase SOCS3 expression nor inhibit the NOS2 mRNA expression and the release of NOx, both in cardiomyocytes and macrophages. Macrophages are crucial in the development of Chronic Chagas Cardiomyopathy. Thus, to deepen our understanding of how Bzl acts, the expression profile of M1-M2 macrophage markers was evaluated. Bzl inhibited the release of NOx (M1 marker) and increased the expression of Arginase I (M2 marker) and a negative correlation was found between them. Besides, LPS increased the expression of pro-inflammatory cytokines. Bzl treatment not only inhibited this effect but also increased the expression of typical M2-macrophage markers like Mannose Receptor, TGF-β, and VEGF-A. Moreover, Bzl increased the expression of PPAR-γ and PPAR-α, known as key regulators of macrophage polarization. PI3K directly regulates M1-to-M2 macrophage polarization. Since p110δ, catalytic subunit of PI3Kδ, is highly expressed in immune cells, experiments were carried out in presence of CAL-101, a specific inhibitor of this subunit. Under this condition, Bzl could neither increase SOCS3 expression nor inhibit NF-κB pathway. Moreover, Bzl not only failed to inhibit the expression of pro-inflammatory cytokines (M1 markers) but also could not increase M2 markers. Taken together these results demonstrate, for the first time, that the anti-inflammatory effect of Bzl depends on PI3K activity in a cell line of murine macrophages and in primary culture of neonatal cardiomyocytes. Furthermore, Bzl-mediated increase expression of M2-macrophage markers involves the participation of the p110δ catalytic subunit of PI3Kδ.

## Introduction

Chagas disease, caused by infection with *Trypanosoma cruzi* (*T. cruzi*), is endemic in several countries of Central and South America and its high morbidity and mortality rates make it a serious public health concern (1). The main approach of pharmacological treatment relies on the use of Benznidazole (*N*-benzyl-2-(2-nitroimidazol-1-yl) acetamide, Bzl), which leads to parasite clearance in the early stages of the disease and contributes to a general immunomodulation (2). In this regard, it has been reported that Bzl not only exerts antiparasitic activity, but also presents anti-inflammatory effects when LPS-challenged murine macrophages are treated with 1mM of Bzl (a high concentration) (3). Moreover, this occurs through the inhibition of the NF-κB pathway (4). Likewise, in LPS-stimulated C57BL/6 mice, high doses of Bzl increase survival and decrease serum levels of IL-6 and TNF-α (5). In a recent work we demonstrated that 15µM of Bzl (a substantial lower dose) can achieve similar anti-inflammatory effects, and make the parasite DNA almost undetectable by qPCR, in an *in vitro* model of murine cardiomyocytes (6). Moreover, we demonstrated that these effects occur through the STAT3/SOCS3 pathway in an IL-10 dependent manner (7). On the other hand, recent works have shown that high doses of Bzl induce the endogenous antioxidant system, increasing antioxidant enzymes expression through NRF2 in an *in vivo* model of sepsis (8) or in an hepatic cell line (9), respectively.

*T. cruzi* induces several cardiac alterations in the chronic phase of the disease, including fibrosis and dilated cardiomyopathy, leading to congestive heart failure in patients with Chronic Chagas Cardiomyopathy (CCC). It has been widely reported that the pathogenesis of CCC relies, partly, on chronic immune-mediated myocardial injury, due to a high infiltration of monocytes and their differentiation into macrophages (10–12). In this scenario in which the balance between pro-inflammatory and anti-inflammatory responses seems to play a key role in avoiding the clinical cardiac manifestations of the disease, the immunomodulatory properties of Bzl, both in cardiac and immune cells, become relevant.

Macrophages are the most abundant immune cells in most tissues of mammals and play a critical role in the immune system (13). Since, they have high plasticity and phenotype can vary depending on the microenvironment (14). The broad spectrum of profiles can range from classical activation (M1) to alternative activation (M2). LPS stimulation induces M1 profile, whose main functions are pro-inflammatory actions in the context of a Th1 response. This is characterized by increased expression of pro-inflammatory cytokines like IL-1β, IL-6, IL-17A and TNF-α; reactive nitrogen species through inducible nitric oxide synthase (NOS2), and the consequent NOx release (15). Conversely, M2 macrophage main functions are anti-inflammatory actions, including tissue remodeling, immunoregulation, angiogenesis and promotion of a Th2 response. These macrophages increase the expression of TGF-β, VEGF-A, CD163, Mannose Receptor and Arginase I (15).

In terms of regulation of macrophage polarization, some key signal regulator factors were identified, such as NF-κB or IRF5 favoring the M1 profile and STAT3, SOCS3, IRF4 and Peroxisome proliferator-activated receptors (PPAR) in favor of the M2 profile (16). PPARs, as main regulators of metabolism, collaborate in the differentiation and expansion of several immune cell types (17). It is widely reported that PPAR-γ plays a major role in macrophage differentiation. Although murine macrophages do not express substantial amounts of PPAR-α, it has been reported that it appears to potentiate the M2 polarization of macrophages.

The regulation of Arginase I expression is also carried out by Phosphatidylinositol 3-kinase (PI3K)/PTEN activity since deletion of PTEN induce M2 polarization *via* STAT3 (18). PI3K activation phosphorylates and activates protein kinase B (PKB, also known as AKT), a family of serine/threonine-specific protein kinases, which downstream effects include the phosphorylation of mTOR and Ribosomal protein S6 kinase beta-1 (mainly known as p70S6K) (19). PI3K/Akt/mTOR pathway is involved in major cellular processes, such as metabolism, cell proliferation, motility and survival (20), and immune system (21).

Class IA PI3K family includes 3 isoforms (PI3Kα, PI3Kβ, PI3Kδ), and has major roles in immune cells. Class IB PI3K includes PI3Kγ isoform (22, 23). The p110α and p110β catalytic subunits of PI3Kα and PI3Kβ respectively, are ubiquitously expressed, while p110δ and p110γ are associated with immune cells and cardiac cells, respectively (24, 25). Although it has been reported that TLR regulation through PI3K is mediated by catalytic subunits as p110β (26) and p110γ (27, 28), several works also reported the role of p110δ in regulation of TLR-4, TLR-3, or TLR-2, by inhibiting the release of pro-inflammatory proteins and increasing IL-10 expression in macrophages and dendritic cells (29–31).

Chagas disease is considered an inflammatory cardiomyopathic syndrome, which includes remodeling, fibrosis, loss of myocardial elasticity and blood supply, ultimately leading to overall cardiac dysfunction (12). The use of drugs that promote repair processes and reduce inflammation could be essential to delay or prevent long-term myocardial damage caused by infection. Deepening the knowledge of the mechanisms of action of the anti-inflammatory effects of Bzl may represent a new step towards a more rational approach to the treatment of Chagas disease. To aim this, in this work we analyzed whether PI3K is involved in the anti-inflammatory properties of Bzl, both in a neonatal mouse primary cardiomyocytes culture and in a cell line of murine macrophages.

## Methods

### Ethics Statement

To carry out this work, CF1 mice were used. All the animals were bread and maintained in the animal facility at the Instituto de Investigaciones en Microbiología y Parasitología Médica (IMPaM, UBA-CONICET), Departamento de Microbiología, Parasitología e Inmunología, Facultad de Medicina. Universidad de Buenos Aires. All the procedures were approved by the Institutional Committee for the Care and Use of Laboratory Animals (CICUAL, Facultad de Medicina de la Universidad de Buenos Aires, RES N° 624/2020), in line with guidelines of the Argentinian National Administration of Medicines, Food and Medical Devices (ANMAT), Argentinian National Service of Agri-Food Health and Quality (SENASA) and with the Guide for the Care and Use of Laboratory Animals (NIH, USA).

### *In Vitro* Models and Stimulation

#### Neonatal Mouse Primary Cardiomyocytes Culture

For each experiment, 20-30 neonatal mice (One- to three-day old, male and female, 2-3g weight) were euthanized by decapitation after CO_2_ exposure in line with the American Veterinary Medical Association (AVMA) guidelines for the euthanasia of animals (32) and cardiomyocytes were obtained as described previously (6, 7). Briefly, the hearts were removed aseptically, pooled and maintained in Hank’s Buffer Saline Solution (HBSS). The hearts were mechanically disaggregated, and the cells were isolated after several digestions with porcine pancreas trypsin (0.25% w/v in PBS). The cells were plated in culture plates in order to generate 5 replicates *per* experimental group, with complete medium (10% FBS-DMEM-M199-PenStrep^®^) at 37°C in a 5% CO_2_ atmosphere, up to 80% confluence. Then, the cells were maintained with 1% FBS-DMEM-M199-PenStrep^®^, at least for a day before the experiments were performed.

#### Murine Macrophage Cell Line RAW 264.7

The murine macrophage cell line RAW 264.7 was cultured in 75 cm^2^ flasks with RPMI medium 1640 (Gibco™) supplemented with 2 mM glutamine, 5% Fetal Bovine Serum (FBS), and PenStrep^®^. Cells were plated in 96-well or 6-well cell culture plates, to generate 5 replicates *per* experimental group, with complete medium (5% FBS-RPMI-PenStrep^®^) at 37°C in a 5% CO_2_ atmosphere, up to 80% confluence. Then, cells were maintained with 1% FBS-RPMI-PenStrep^®^, at least for a day until use.

#### *In Vitro* Stimulation and Treatment

**Figure 1:**
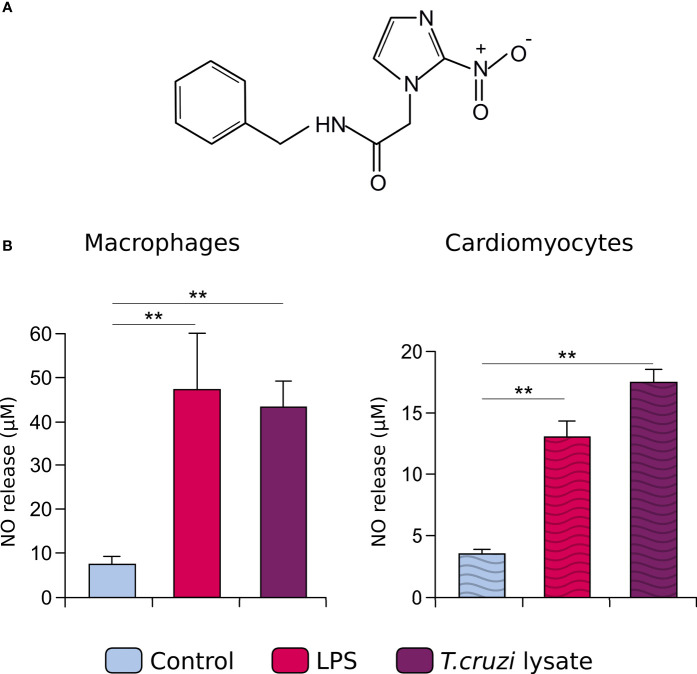
The chemical structure of benznidazole (Bzl), the drug used in this study is shown **(A)**. RAW 264.7 macrophages and neonatal primary cardiomyocytes were treated with 15 µM of Bzl for 30 minutes and then with 10 µg/mL of LPS or 10 µg/mL of *T. cruzi* lysate. After 48 h, NO levels were quantified by the Griess reaction in culture supernatants **(B)**. Results are expressed as the mean of 3 independent experiments (n=3, 5 replicates/treatment) ± SEM. **P < 0.001 vs. control cells.

Bzl (Abarax^®^, ELEA, Argentina. PubChem Compound Database CID=31593, **Figure 1A**) was suspended in PBS. Cells were pre-treated with Bzl 15 µM for 30 minutes before stimulation.

Stimulations with LPS (10 µg/mL) from *Escherichia coli* O55:B5 (Santa Cruz Biotechnologies) were performed for the indicated period, in polystyrene culture plates.

To verify that the LPS stimulation produces an inflammatory response like that generated by parasite antigens, the cells were stimulated with 10 µg/mL of a *T. cruzi* trypomastigote lysate from the RA *T. cruzi* strain. Vero cell line was cultured in cell culture bottles of 175 cm^2^ with RPMI supplemented with 10% fetal bovine serum (FBS), 100 units/mL Penicillin, 0.1 mg/mL Streptomycin and 2mM L-glutamine. When culture reached an approximate 50% confluence, it was infected with parasites of the RA strain of *T. cruzi.* After 6 h, culture medium was washed to remove non-infective parasites and cells were incubated at 37°C for 48 h. On day 5 post-infection, trypomastigotes were harvested from the supernatant. The culture medium was collected, two washes were performed with cold PBS and the parasite pellet was stored at -80°C. After one month of collection, all sediments were pooled and lysed to obtain trypomastigote proteins. Briefly, sediments were resuspended in lysis buffer (PBS, 10 µM E-64 and 3 µg/mL protease inhibitor) and subjected to 3 freeze/thaw cycles (-80°C/room temperature) of 30 min each. Then, the pool was incubated overnight at -80°C and centrifuged at 17000 xg at 4°C for 10 minutes. The supernatant was collected, and protein concentration was quantified by the Bradford method using a commercial protein assay (Bio-Rad, USA) and bovine serum albumin (BSA) (Sigma-Aldrich Co, USA) as a standard (33) as described previously by our group (7, 34, 35).

#### PI3K Inhibition

First, primary cardiomyocytes and RAW 264.7 macrophages were treated with 30 µM of the specific PI3K inhibitor LY294002 (Santa Cruz Biotechnology, USA) for 30 minutes before Bzl treatment. LY294002 competitively inhibits the ATP binding site of PI3K. This directly affects its activity but does not affect other enzymes that need ATP (36). Briefly, LY294002 was solubilized in DMSO to prepare the 15 mM stock solution. Working solution (100 µM) was made by diluting the stock solution in PBS. Phosphorylation of P70S6K, a serine/threonine kinase that acts downstream of PI3K/AKT/mTOR (19), was evaluated as a hallmark of activation of PI3K pathway and as a control of inhibition.

RAW 264.7 macrophages were treated with 10 µM CAL-101 (Santa Cruz Biotechnology, USA), a specific inhibitor of p110δ catalytic subunit of Class 1A PI3K. Briefly, CAL-101 was solubilized in DMSO to render a 24 mM stock solution. Working solution (100 µM) was made by diluting the stock solution in PBS. Phosphorylation of P70S6K, a serine/threonine kinase that acts downstream of PI3K/AKT/mTOR (19) and phosphorylation of AKT, were evaluated as a hallmark of activation of PI3K pathway and as a control of inhibition.

### RNA Purification

Total RNA was obtained from macrophages using Quick-zol reagent (Kalium Technologies, Argentina), treated with RQ1 RNase-Free DNase (Promega Co., USA). Total RNA was reverse-transcribed using M-MLV Reverse Transcriptase (Promega Co., USA), according to manufacturer’s instructions, as previously described (7, 35). Purity of extracted RNA was checked by calculation of the ratios of spectrophotometric absorbance of the sample, both A_260_/A_280_ and A_260_/A_230_. Extracted RNA was considered pure when A_260_/A_280_ >2.0 (since lower ratios indicate that sample is contaminated by protein or phenol residues) and 2.0>A_260_/A_230_ >2.2 (since lower ratios indicate that sample is contaminated by phenol residues).

### Quantitative Reverse Transcription Polymerase Chain Reaction (RT-qPCR)

mRNA expression analysis was performed using 5X HOT FIREPOL EVAGREEN qPCR (Solis BioDyne, Estonia) in a StepOnePlus Real-Time PCR System. Parameters were: 52°C for 2 min, 95°C for 15 min, and 40 cycles at 95°C for 15 s, specific Tm °C for 30 s and 72°C for 1 min. Normalization was carried out using β-actin mRNA. This pair of primer sequences was used by several authors to normalize gene expression in both RAW 264.7 macrophages (37–39) and cardiac cells (40–42). Primer sequences used for RT-qPCR, including melting temperature and amplicon size, were shown in **Table 1**. Quantification was performed using the comparative threshold cycle (Ct) method, as all the primer pairs (target gene/reference gene) were amplified using comparable efficiencies (relative quantity, 2^-ΔΔCt^) (43, 44).

**Table 1:** Primer sequences used for RT-qPCR, including melting temperature and amplicon size, were provided.

	Forward (5’-3’)	Reverse (5’-3’)	Tm (°C)	Amplicon size (bp)
β-actin:	GGCTGTATTCCCCTCCATCG	CCAGTTGGTAACAATGCCATGT	62	241
IL-6:	TGATGCACTTGCAGAAAACAA	GGTCTTGGTCCTTAGCCACTC	60	270
IL-17A:	GAAGGCAGCAGCGATCATC	CGTTTCCCTCCGCATTGACA	65	255
MR:	CAAGGAAGGTTGGCATTTGT	CCTTTCAGTCCTTTGCAAGC	60	111
NOS2:	CACAGCAATATAGGCTCATCCA	GGATTTCAGCCTCATGGTAAAC	60	101
PPAR-α:	CCATACAGGAGAGCAGGGATTT	TTACCTACGCTCAGCCCTCTTC	62	76
PPAR-γ:	ATCTACACGATGCTGGC	GGATGTCCTCGATGGG	60	253
SOCS3:	CCTTTGACAAGCGGACTCTC	CCTTTGACAAGCGGACTCTC	60	216
TNF-α:	CGGGCAGGTCTACTTTGGAG	ACCCTGAGCCATAATCCCCT	62	166
VEGF-A:	TGCGGGATCAAACCTCACCA	TCTCCGCTCTGAACAAGG	62	453

bp, base pairs.

### Protein Extraction and Western Blot Analysis

Cultured cells were washed with PBS and scraped off the dishes, with 50 μL of the Buffer A (10 mmol/L HEPES pH=7.90, 1 mmol/L EDTA, 1 mmol/L EGTA, 10 mmol/L KCl, 1 mmol/L DTT, 0.5 mmol/L PMSF, 40 mg/L leupeptin, 2 mg/L tosyl-lysyl-chloromethane, 5 mmol/L NaF, 1 mmol/L NaVO_4_, 10 mmol/L Na_2_MoO_4_), and NP-40 (Life Technologies) was added to reach 0.5% (v/v). After 15 min at 4°C, the tubes were gently vortexed for 10 s, and cytosolic extracts were collected by centrifugation at 13,000 xg for 90 s. Supernatants were stored at −20°C (cytosolic extracts), and pellets were resuspended in 100 μL buffer A supplemented with 20% (v/v) glycerol and 0.4 M KCl, and mixed for 30 min at 4°C. Nuclear proteins were obtained by centrifugation at 13,000 xg for 5 min, and aliquots of the supernatant (nuclear extracts) were stored at −80°C.

Total protein extracts were obtained after washing the cultured cells with PBS and scraped off the dishes with 50 uL of the RIPA modified lysis buffer (150 mM NaCl, 50 mM Tris-HCl (pH=7.40), 1% Triton X-100, 1mM). Then, the tubes were kept on ice for 30 min with swirling and the samples were centrifuged at 7000 xg at 4°C for 10 min. The supernatant was stored at −20°C (7, 35).

Protein concentration was determined by the Bradford method using a commercial protein assay (Bio-Rad, USA) and bovine serum albumin (BSA) (Sigma-Aldrich Co, USA.) as a standard (33).

Fifty µg of protein extracts separated in 8-12% SDS-PAGE gels were blotted onto a Hybond-P membrane (GE Health-care, Spain) to detect p-AKT-Ser473 (Cat#649001, Biolegend Inc), total AKT (Cat#680302 Biolegend Inc), p-P70S6K-Ser434 (Cat#sc-8416, Santa Cruz Biotechnology), total P70S6K (Cat#sc-8418, Santa Cruz Biotechnology), SOCS3 (Elabscience^®^, Cat#E-AB-10603), Arginase I (Cat#sc-20150, Santa Cruz Biotechnologies), IκBα (Cat#sc-1643, Santa Cruz Biotechnologies), p65 (Cat# sc-514451) and β-actin (Cat#sc-8432, Santa Cruz Biotechnologies), using specific antibodies in a 1:500 dilution in PBS. To ensure the same protein load in each lane, we prepared a sample solution containing 2 µg/µL final protein concentration and 25 µL of this solution was loaded onto the gel.

Mouse anti-rabbit IgG-HRP (Cat#sc-2357, Santa Cruz Biotechnologies) or Mouse IgGκ light chain binding protein, m-IgGκ BP-HRP, (Cat#sc-516102, Santa Cruz Biotechnologies) were used for antibody detection in a 1:3000 dilution in PBS.

Blots were revealed using enhanced chemiluminescence (ECL) horseradish peroxidase (HRP) substrate (Thermo Scientific SuperSignal™ West Pico PLUS Chemiluminescent Substrate) in a BioSpectrum^®^ Imaging Systems (UVP, Analytik Jena Company, USA).

Normalized relative density score were calculated for the western blot bands using the Image J software (NIH, USA). Briefly, in the first place we calculated the normalized signal for all target proteins, calculating the ratio of the signal intensity of the target band in each lane to the signal intensity of β-actin, total-P70S6K or total-AKT, as appropriate. Then, the relative band intensity was determined as the ratio of the experimental sample normalized signal to the control sample normalized signal (45).

### NOx Measurement

To determine the amount of NOx released into the culture medium, nitrite was measured spectrophotometrically by the Griess reaction (46, 47). Absorbance at 540 nm was compared with a standard curve of NaNO_2_.

### Statistical Analysis

Data are expressed as the mean of three independent experiments ± SEM (n=3 for each treatment, five culture replicates/group). One-way ANOVA was used to analyze the statistical significance of the differences observed between infected, treated, or untreated groups. The Tukey *post-hoc* test was carried out to compare every mean with every other mean. The Pearson rank-order correlation test was used to evaluate the correlation between NOx levels and Arginase I expression (Power = 0.8). Differences were considered statistically significant when *P*<0.05. All analyses were performed using the Prism 7.0 Software (GraphPad, USARRID:SCR_002798).

## Results

Since it has been reported that PI3K pathway is involved in the regulation of the immune system this work aims to evaluate whether PI3K participates in Bzl effects.

### Role of the PI3K Pathway in the Anti-Inflammatory Effect of Bzl in Murine Macrophages and Cardiomyocytes

To assess the involvement of PI3K in Bzl effects on both immune and cardiac cells, we used a murine macrophage cell line (RAW 264.7) and a murine primary culture of cardiomyocytes, respectively. To unlink the anti-inflammatory effects of Bzl from its parasiticidal effect, cells were pretreated with Bzl and then stimulated with LPS in lieu of parasites, since LPS is recognized by TLR4 like some *T. cruzi* components, both increasing the expression of pro-inflammatory mediators. To confirm that and to validate the stimulation model, we decided to evaluate the inflammatory response in terms of the release of NOx to the culture supernatant, in both stimulation with LPS or a *T. cruzi* lysate enriched in parasite proteins. We found that both stimuli increased the amount of NOx in the culture supernatant, without significant differences between them (**Figure 1B**).

**Figure 2:**
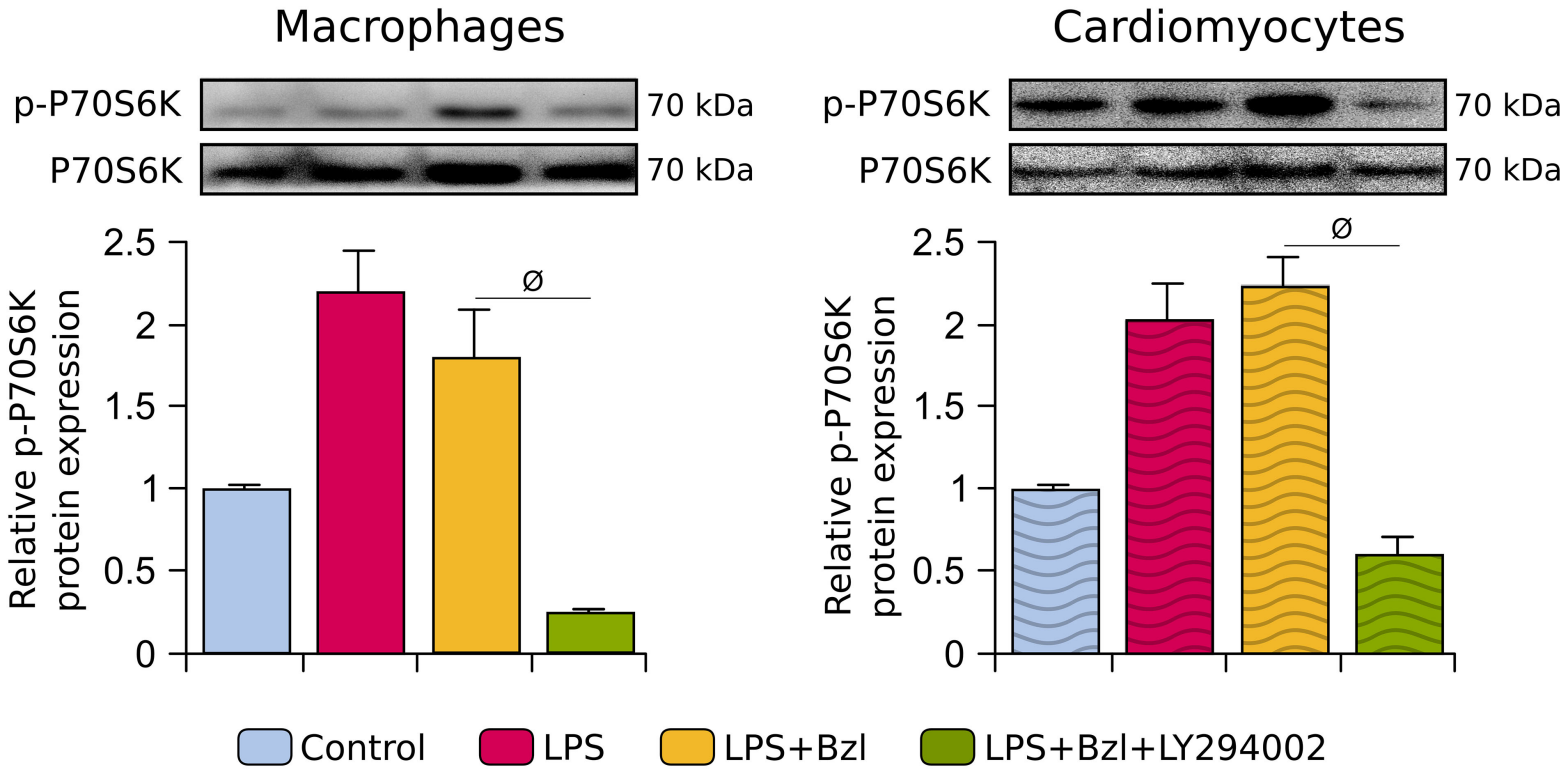
RAW 264.7 macrophages and neonatal primary cardiomyocytes were treated with 30 μM LY294002, a non-isoform-selective inhibitor of PI3K, 30 min before the addition of 15 μM benznidazole (Bzl). Cells were stimulated with 10 µg/mL of LPS 30 min after Bzl treatment. After 30 minutes, relative p-P70S6K (70kDa) protein expression was determined by Western blot with a specific antibody and normalized against total P70S6K (70kDa). Results are expressed as the mean of 3 independent experiments (n=3, 5 replicates/treatment) ± SEM. *^ϕ^P < 0.05 vs*. Bzl-treated and LPS-stimulated cells.

Then, for the first approach, RAW 264.7 macrophages and primary cardiomyocytes were pre-treated with 15 µM of Bzl and stimulated with LPS, in presence of a non-isoform-selective inhibitor of PI3K (LY294002) (48). Afterwards, the phosphorylation of P70S6K, a serine/threonine kinase that acts downstream of PI3K/AKT/mTOR (19), was evaluated as a hallmark of activation of PI3K pathway. As expected, treatment with LY294002 inhibited the phosphorylation of P70S6K in both cell types (**Figure 2**).

**Figure 3:**
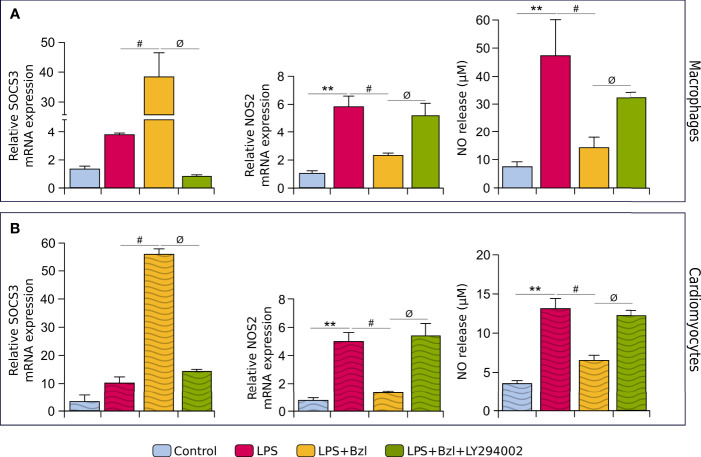
RAW 264.7 macrophages **(A)** and neonatal primary cardiomyocytes **(B)** were treated with 30 μM LY294002, a non-isoform-selective inhibitor of PI3K, 30 min before the addition of 15 μM benznidazole (Bzl). Cells were stimulated with 10 µg/mL of LPS 30 min after Bzl treatment. After 48 h, NOS2 mRNA levels was analyzed by RT-qPCR and normalized against β-actin mRNA and NOx levels were quantified by the Griess reaction in culture supernatants. After 24 h, SOCS3 mRNA level was analyzed by RT-qPCR and normalized against β-actin mRNA. Results are expressed as the mean of 3 independent experiments (n=3, 5 replicates/treatment) ± SEM. **P < 0.001 vs. control cells, *^#^P < 0.05 vs* LPS-stimulated cells. *^ϕ^P < 0.05 vs.* Bzl-treated and LPS-stimulated cells.

Since we have previously described that Bzl inhibits NF-κB activity by increasing SOCS3 expression (7), we decided to evaluate whether PI3K was involved. Here we found that in the presence of LY294002, Bzl failed to increase SOCS3 and thus, also failed to inhibit the inflammatory response in terms of NOS2 mRNA expression and NOx release to the culture supernatant, in both macrophages (**Figure 3A**) and cardiomyocytes (**Figure 3B**). These findings suggest that Bzl-mediated anti-inflammatory effects depend on the activity of PI3K.

### Benznidazole Modifies the Expression Profile of M1-M2 Markers in Macrophages

In order to deep into the knowledge on Bzl mechanism of action and considering that macrophages play a key role in the pathogenesis of CCC, we decided to study the expression profile of M1-M2 markers.

**Figure 4:**
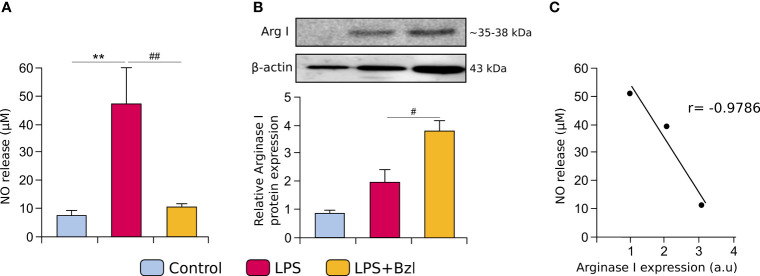
RAW 264.7 macrophages were treated with 15 µM of Bzl for 30 minutes and then with 10 µg/mL of LPS. After 48 h, NOx levels were quantified by the Griess reaction in culture supernatants **(A)** and relative Arginase I (~35-38 kDa) protein expression was determined by Western blot with a specific antibody and normalized against β-actin (43 kDa) **(B)**. Relationship analysis between NOx release and Arginase I expression was performed using a Pearson rank-order correlation test and Pearson correlation coefficient (r) is reported (Power=0.8) **(C)**. Results are expressed as the mean of 3 independent experiments (n=3, 5 replicates/treatment) ± SEM. **P < 0.001 vs. control cells; *^#^P < 0.05, ^##^P < 0.001 vs* LPS-stimulated cells.

**Figure 4** shows that LPS significantly increases the release of NOx to culture supernatant and does not significantly modify Arginase I expression. Bzl not only inhibits NOx release (**Figure 4A**) but also significantly increases Arginase I expression (**Figure 4B**) with a negative correlation between these effects (**Figure 4C**).

**Figure 5:**
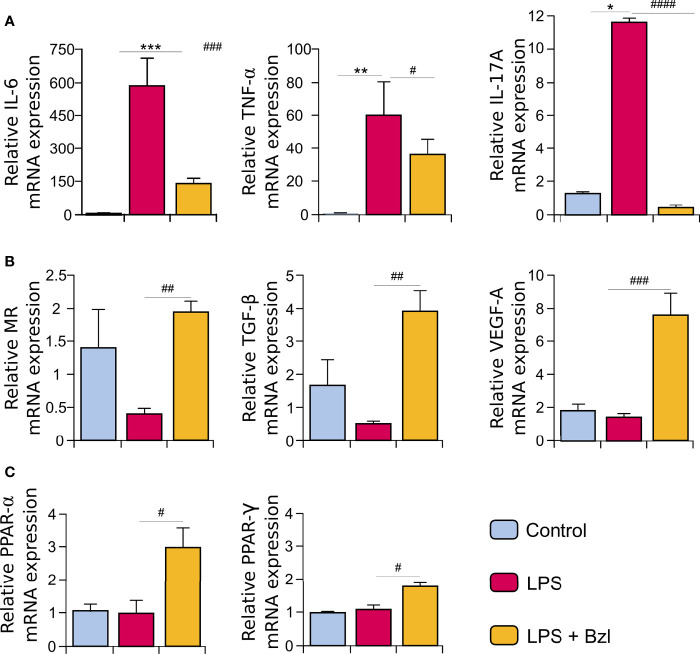
RAW 264.7 macrophages were treated with 15 µM of benznidazole (Bzl) for 30 minutes and then with 10 µg/mL of LPS. After 4 h, IL-6, TNF-α and IL-17A mRNA levels were analyzed by RT-qPCR and normalized against β-actin mRNA **(A)**. After 24 h, Mannose Receptor (MR), TGF-β, VEGF-A **(B)**, PPAR-α and PPAR-γ **(C)** mRNA levels were analyzed by RT-qPCR and normalized against β-actin mRNA. Results are expressed as the mean of 3 independent experiments (n=3, 5 replicates/treatment) ± SEM. *P < 0.05, **P < 0.001, ***P < 0.0001 vs. control cells; *^#^P < 0.05, ^##^P < 0.001, ^###^P < 0.0001, ^####^P < 0.00001 vs* LPS-stimulated cells.

To continue the characterization of the expression profile of Bzl-treated macrophages, mRNA expression of M1- and M2-macrophage markers was evaluated. **Figure 5A** shows that Bzl inhibits IL-6, TNF-α and IL-17A mRNA expression, typically M1-macrophage mediators. On the contrary, Bzl increases mRNA expression of the surface marker Mannose Receptor (MR), and growth factors like TGF-β and VEGF-A (**Figure 5B**), typical M2-macrophage markers (16, 49). It has been reported that the acquisition and long-term maintenance of M2-like phenotype requires metabolic regulators such as PPAR-γ (50, 51), whose expression, as well as that of PPAR-α, is increased in Bzl-treated macrophages (**Figure 5C**).

### Benznidazole Inhibits NF-κB Activation Through SOCS3 in a PI3Kδ Dependent Manner

**Figure 6:**
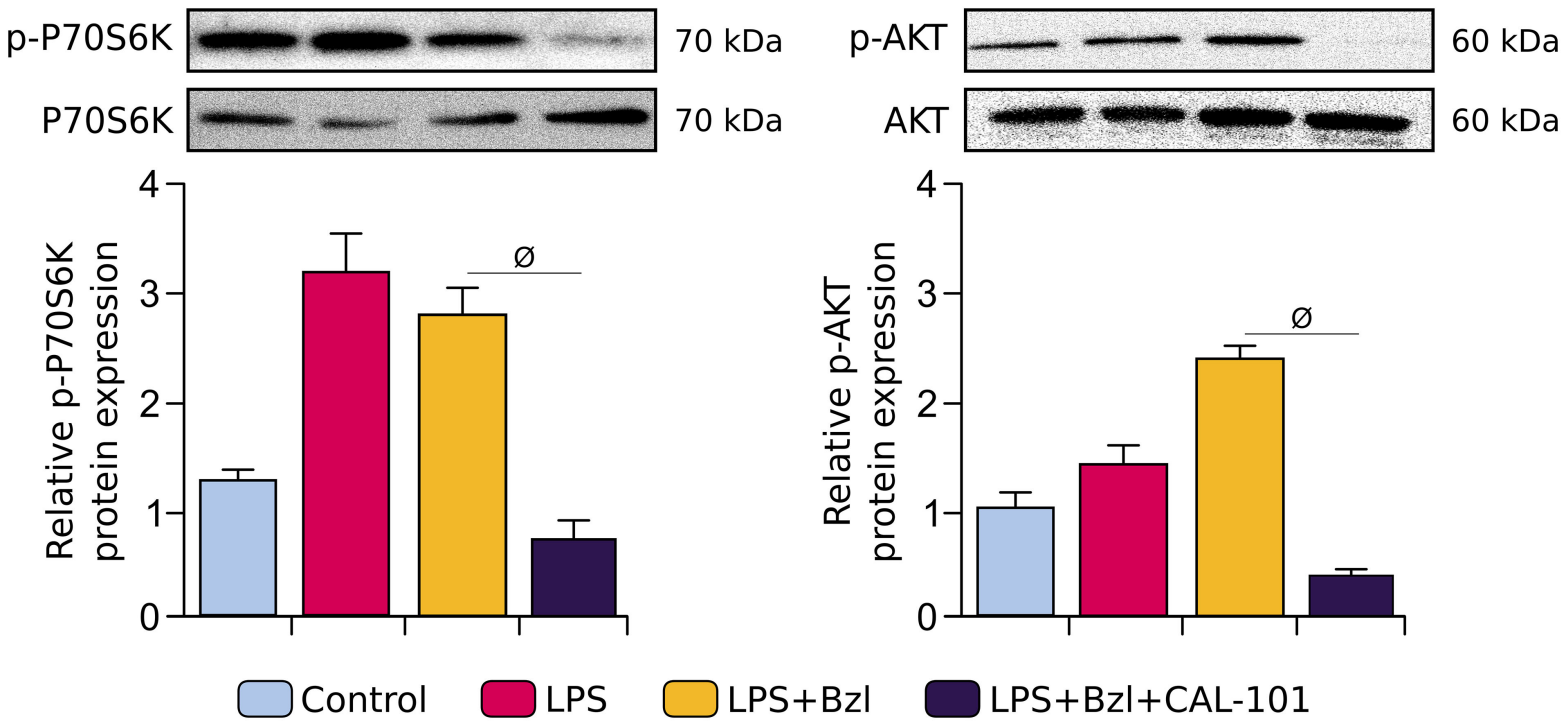
RAW 264.7 macrophages were treated with 10 μM CAL-101, a specific inhibitor of p110δ, the catalytic subunit of PI3Kδ isoform, 30 min before the addition of 15 μM benznidazole (Bzl). Cells were stimulated with 10 µg/mL of LPS 30 min after Bzl treatment. After 30 minutes, relative p-P70S6K (70 kDa) and relative p-AKT (60 kDa) protein expression was determined by Western blot with a specific antibody and normalized against total P70S6K (70 kDa) and AKT (60 kDa), respectively. Results are expressed as the mean of 3 independent experiments (n=3, 5 replicates/treatment) ± SEM. *^ϕ^P < 0.05 vs.* Bzl-treated and LPS-stimulated cells.

PI3K Class IA has major functions in the immune system and includes different isoforms according to the catalytic subunit involved (22). Since LY294002 is a non-isoform-selective inhibitor of PI3K, and to deepen the knowledge on the role of PI3K in Bzl effects, it was interesting to study which PI3K isoforms were implicated. Since p110δ, catalytic subunit of PI3Kδ isoform, is expressed in immune system cells and it can regulate inflammatory response (52), RAW 264.7 macrophages were pre-treated with Bzl and stimulated with LPS in presence of CAL-101, a specific inhibitor of this subunit (34). Phosphorylation of P70S6K and AKT was evaluated as an assay marker of the activation of the PI3K pathway. As expected, treatment with CAL-101 inhibits both the phosphorylation of P70S6K and AKT (**Figure 6**).

**Figure 7:**
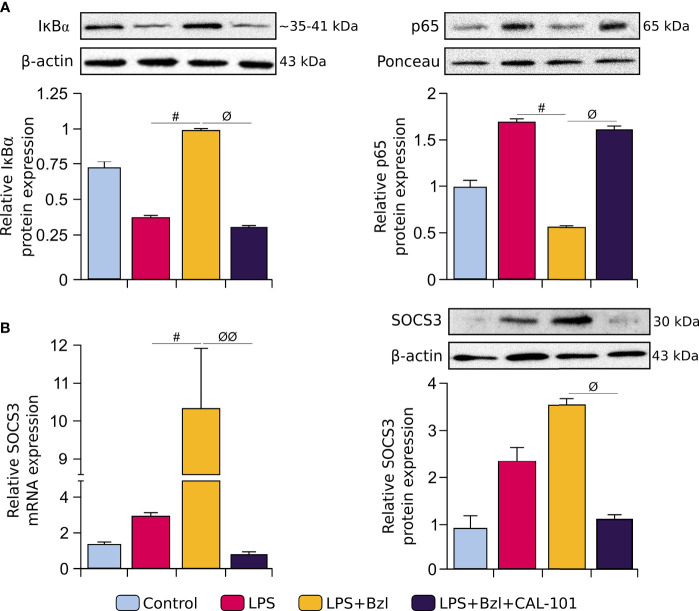
RAW 264.7 macrophages were treated with 10 μM CAL-101, a specific inhibitor of p110δ, the catalytic subunit of PI3Kδ isoform, 30 min before the addition of 15 μM benznidazole (Bzl). Cells were stimulated with 10 µg/mL LPS 30 min after Bzl treatment. After 30 minutes, relative cytosolic IκBα (~35-41 kDa) and nuclear p65 (65 kDa) protein expression was determined by Western blot with a specific antibody and normalized against β-actin and Ponceau-staining, respectively **(A)**. After 24 h, SOCS3 mRNA levels were analyzed by RT-qPCR and normalized against β-actin mRNA and after 48 h, relative SOCS3 (25 kDa) protein expression was determined by Western blot with a specific antibody and normalized against β-actin (43 kDa) **(B)**. Results are expressed as the mean of 3 independent experiments (n=3, 5 replicates/treatment) ± SEM. *^#^P < 0.05 vs* LPS-stimulated cells. *^ϕ^P < 0.05, ^ϕϕ^P < 0.001 vs.* Bzl-treated and LPS-stimulated cells.

To evaluate whether p110δ was implicated in Bzl anti-inflammatory effect, we analyzed the cytosolic expression of IκBα and nuclear p65, in stimulated and treated macrophages. In presence of CAL-101, Bzl did not inhibit neither the cytosolic degradation of IκBα nor the nuclear translocation of p65, indicating that p110δ is required for Bzl-mediated inhibition of NF-κB (**Figure 7A**). Besides, when p110δ was inhibited, Bzl could not increase SOCS3 expression by both RT-qPCR and WB (**Figure 7B**).

### Benznidazole Increases M2 Macrophage Markers Through p110δ Catalytic Subunit of PI3Kδ

**Figure 8:**
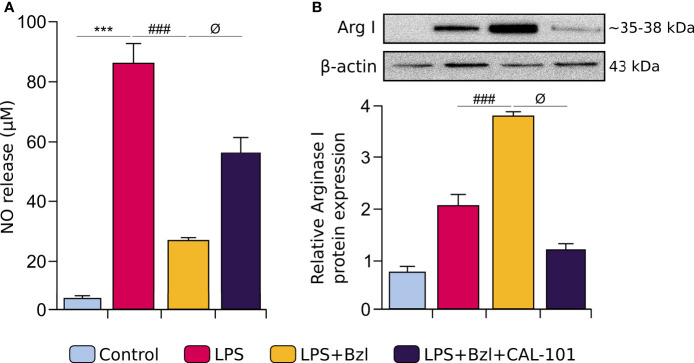
RAW 264.7 macrophages were treated with 10 μM CAL-101, a specific inhibitor of p110δ, the catalytic subunit of PI3Kδ isoform, 30 min before the addition of 15 μM benznidazole (Bzl). Cells were stimulated with 10 µg/mL LPS 30 min after Bzl treatment. After 48 h, NO levels were quantified by the Griess reaction in culture supernatants **(A)** and relative Arginase I (~35-38 kDa) protein expression was determined by Western blot with a specific antibody and normalized against β-actin (43 kDa) **(B)**. Results are expressed as the mean of 3 independent experiments (n=3, 5 replicates/treatment) ± SEM. ***P < 0.0001 vs. control cells, ^###^ *P < 0.0001 vs* LPS-stimulated cells. ^ϕ^ *P* < 0.05 *vs*. Bzl-treated and LPS-stimulated cells.

In this work we demonstrated that Bzl not only inhibits NOx release but also significantly increases Arginase I expression. Therefore, it was intriguing to know whether these effects depend on the p110δ subunit of PI3K. **Figure 8** shows that in presence of CAL-101, Bzl was unable to inhibit NOx release (**Figure 8A**) or increase Arginase I expression (**Figure 8B**).

**Figure 9:**
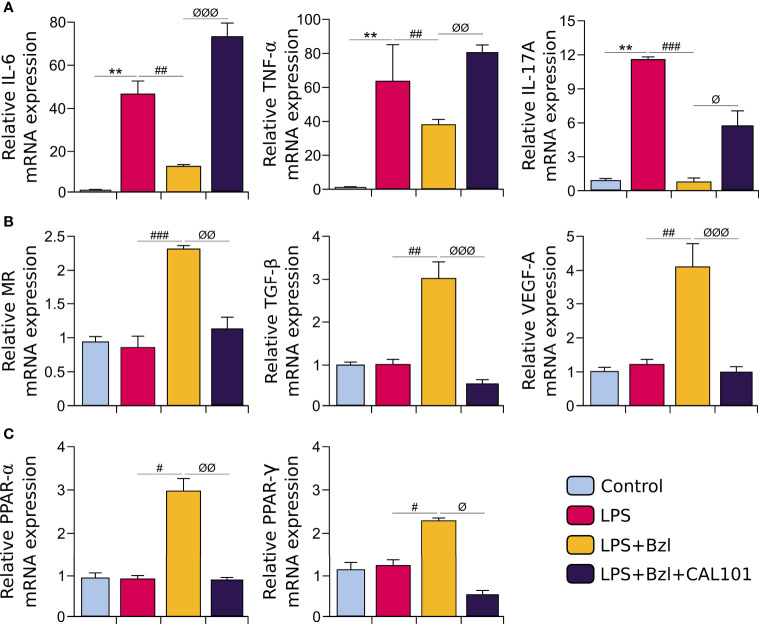
RAW 264.7 macrophages were treated with 10 μM CAL-101, a specific inhibitor of p110δ, the catalytic subunit of PI3Kδ isoform, 30 min before the addition of 15 μM benznidazole (Bzl). Cells were stimulated with 10 µg/mL LPS 30 min after Bzl treatment. After 4 h, IL-6, TNF-α and IL-17A mRNA levels were analyzed by RT-qPCR and normalized against β-actin mRNA **(A)**. After 24 h, Mannose Receptor (MR), TGF-β, VEGF-A **(B)**, PPAR-α and PPAR- γ **(C)** mRNA levels were analyzed by RT-qPCR and normalized against β-actin mRNA, and after 48 h relative Arginase I protein expression was determined by Western blot with a specific antibody and normalized against β-actin **(B)**. Results are expressed as the mean of 3 independent experiments (n=3, 5 replicates/treatment) ± SEM. **P < 0.001 vs. control cells; *^#^P < 0.05, ^##^P < 0.001, ^###^P < 0.0001 vs* LPS-stimulated cells*. ^ϕ^P < 0.05, ^ϕϕ^P < 0.001, ^ϕϕϕ^P < 0.0001 vs.* Bzl-treated and LPS-stimulated cells.

As expected, the anti-inflammatory effect of Bzl was precluded by CAL-101, since Bzl was unable to inhibit mRNA expression of pro-inflammatory cytokines as TNF-α, IL-6 and IL-17A evaluated by RT-qPCR (**Figure 9A**). Moreover, Bzl was unable to increase the expression of MR, TGF-β, VEGF-A (**Figure 9B**), PPAR-α and PPAR-γ mRNA expression (**Figure 9C**), evidencing that p110δ subunit of PI3K is required to Bzl-mediated increase of M2 macrophage markers.

In summary these results show, for the first time, that the anti-inflammatory effect exerted by Bzl in murine macrophages and cardiomyocytes is mediated by PI3K. Furthermore, we demonstrated that Bzl reduced M1-macrophages marker expression and increased M2 macrophage markers expression, in a Class I PI3K δ dependent manner.

## Discussion

Benznidazole (Bzl) is a derivative of 2-nitroimidazole and is one of the nitroheterocyclic compounds used against parasitic diseases (53). Even though Bzl and its antiparasitic activity were found more than 40 years ago (54) and that it presents several adverse effects (55), it remains as the first-line treatment for Chagas disease worldwide (56). The therapeutic action of Bzl is based on the elimination of the etiological agent, *T. cruzi* (57), but, in addition to its antiparasitic effects, it has also been reported to exert immunomodulatory effects favoring a balanced, non-exacerbated, inflammatory response in Chagas disease patients (2).

In a previous work we described that Bzl maintains its parasiticidal properties at a lower dose (25 mg/Kg/day) or concentration (15 µM) than those previously reported for a highly virulent *T. cruzi* strain (6). Moreover, in this *in vivo* model of infection, daily treatment (orally administered) with 25 mg/kg/day Bzl induced a significant decrease in IL-1β, IL-6 and NOS2 in the heart and CK activity in serum, to normal levels (6). No mortality was observed in infected treated mice. Furthermore, Bzl prevents the production of inflammatory mediators by inhibiting the NF-κB inflammatory pathway in the *in vitro* model of murine cardiomyocytes (6).

Prolonged inflammation in the myocardium induces irreversible structural and functional alterations that may lead to CCC (58, 59). Therefore, the study of cardiac inflammation, as well as the research for treatments to control it, becomes essential. Since macrophages participate in the development of CCC (60), we aimed to study how Bzl acts both in cardiac cells and macrophages. To this aim, we used a neonatal primary cardiomyocytes culture and a RAW 264.7 cell line. To explore the anti-inflammatory effects of Bzl, independently of its anti-parasitic effects, cells were stimulated with LPS since it is recognized by TLR4 similarly to *T. cruzi* components, such as glycoinositolphospholipids (61, 62). In this regard, we first confirmed that LPS increases NOx release in the same way that *T. cruzi* lysate enriched on parasite proteins.

The PI3K pathway not only has a critical role in the modulation of pro-inflammatory response (63) but also directly regulates M1-to-M2 macrophage polarization (28, 64). Thus, to increase our knowledge regarding the mechanism of action implicated in Bzl effects, we aimed to study whether this signaling pathway was involved. When analyzing the phosphorylation of the kinase p70S6, a component found downstream of the PI3K pathway, we observed that stimulation with LPS tends to increase the phosphorylation of this protein in comparison with the control. These results are in line with other authors who found that PI3K was triggered after activation of TLR in macrophages to negatively regulate the inflammatory response (28, 65).

We have previously described, in a murine cardiomyocyte model, that Bzl inhibits the NF-κB pathway, at least in part, through the STAT3/SOCS3 pathway in an IL-10-dependent manner (7). Here, we found similar results, since Bzl significantly increases SOCS3 expression and inhibits NF-κB activation and the release of NOx, both in cardiac cells and macrophages. Zhang et al. demonstrated that PI3K/Akt/SOCS3 pathway negatively regulates the inflammatory response in a murine macrophage cell line (RAW 264.7) upon stimulation with lipoteichoic acid (66). In this regard, comparable results were obtained in this work. Although Bzl did not significantly increase the phosphorylation of P70S6K, in comparison with LPS stimulation, the anti-inflammatory effects of Bzl depend on PI3K, at least at a certain degree, since in presence of LY294002, Bzl was unable to exert its immunomodulatory effect in both cell types.

It has been widely reported that macrophages can be alternatively activated in order to maintain homeostasis and regulate the resolution of the inflammatory response (67, 68). This M1/M2 classification can also be summarized as two opposing ways of arginine metabolism: M1 macrophages metabolize it *via* nitric oxide synthase (NOS) to increase NO release, while M2 macrophages metabolize it *via* Arginase I to synthesize ornithine and urea (69). These results show, for the first time, that Bzl not only inhibited the NO release and IL-6, TNF-α and IL-17A mRNA expression (M1 markers) but also increased Arginase I and Mannose Receptor (MR), TGF-β, VEGF-A, PPAR-α and PPAR-γ mRNA expression (M2 markers), suggesting that Bzl modifies the expression profile in macrophages.

It has been reported that the PI3K pathway plays a major role in the regulation of Toll Like Receptors (TLRs) and in the induction of anti-inflammatory effects through SOCS3 in some cell types (66). Moreover, PI3K is known as a negative regulator of NF-kB signaling (65) due to its critical role not only in the inhibition of pro-inflammatory but also in the promotion of anti-inflammatory responses in LPS-stimulated macrophages (70). In fact, inhibition with Rapamycin of mTOR, essential serine-threonine kinase downstream of PI3K, suppresses the phosphorylation of STAT3 and activates NF-κB (71). Several authors have shown the relationship between macrophage polarization and PI3K. For instance, Rocher et al. have demonstrated that BMP-7 polarizes monocytes into M2 macrophages by the activated SMAD-PI3K-Akt-mTOR pathway (72). In addition, Wei et al. recently showed that PD-L1 induces macrophage polarization *via* Erk/Akt/mTOR (73) and Nishikoba *et al* demonstrated that Hepatocyte Growth Factor (HGF)-MET signaling does it through PI3K-mediated induction of Arginase I (74). Class IA PI3K isoforms have a major role in immune system cells. Particularly, PI3Kδ is the most abundant isoform in these cells, and it has been reported to be critical for the activation and proliferation of macrophages attached to the extracellular matrix (75). It has been described that M2-macrophage polarization requires a metabolic switch which depends on the PI3K-AKT-mTOR pathway (76). Although both M1 and M2 macrophages enhance glutamine consumption, it is used and routed into different metabolic pathways. Furthermore, M1 macrophages show increased glycolysis, glucose absorption and the activity of pentose phosphate pathway is increased; on the other hand, M2 macrophages show increased levels of oxidative metabolism and reduced glycolytic rates (77).

In order to evaluate whether PI3Kδ was involved in Bzl effects, RAW 264.7 macrophages were treated with CAL-101, a specific inhibitor of p110δ catalytic subunit of PI3K. Although the role of PI3Kδ remains unclear, it has been reported that the lack of this isoform in LPS-stimulated dendritic cells, reduces the internalization of TLR4 and its relocation in endosomes. This increases the secretion of pro-inflammatory cytokines like IL-6 and IL-12 in the early stage, and reduces the release of anti-inflammatory cytokines such as IL-10 and IFN-β in the late stage (31). In this work we show, for the first time, that PI3Kδ participates in Bzl effects, since CAL-101 precluded the inhibition of NF-κB and the increase in SOCS3 expression. Moreover, under this condition, Bzl could neither inhibit the NO release, IL-6, TNF-α or IL-17A mRNA expression (M1 markers), nor promote the increase in Arginase I and Mannose Receptor (MR), TGF-β, VEGF-A, PPAR-α or PPAR-γ mRNA expression (M2 markers), suggesting that Bzl-mediated M1-to-M2 expression profile in macrophages depends on PI3Kδ. In this regard, several works have shown the role of p110δ in the regulation of TLR-2, TLR-3 and TLR-4 (29–31).

Chagas disease is considered an inflammatory cardiomyopathic syndrome (12) and Bzl is one of the few available drugs for its treatment. This is the reason why several ongoing clinical trials are aimed to find new treatment regimens with Bzl. For instance, the BENDITA trial recently published preliminary results showing that shorter or reduced regimens of Bzl can reach similar results in terms of parasite clearance but with greater safety (78). Increasing the understanding of Bzl mechanism of action is a necessary step in order to take advantage of its anti-inflammatory effects.

The results presented in this work demonstrate, for the first time, that Bzl anti-inflammatory effects depend on PI3K, both in cardiac cells and macrophages. Furthermore, Bzl modifies the profile expression toward a M2-type in a PI3Kδ dependent manner.

This knowledge may represent a new step towards a more rational approach to Chagas disease treatment.

## Data Availability Statement

The original contributions presented in the study are included in the article/supplementary material. Further inquiries can be directed to the corresponding authors.

## Ethics Statement

The animal study was reviewed and approved by Comisión Institucional para el Cuidado y Uso de Animales de Laboratorio (CICUAL), Facultad de Medicina, Universidad de Buenos Aires.

## Author Contributions

ÁC and NG designed experiments. ÁC, PM, AP, AS, and FP did experiments. ÁC, PM, NG, and GM contributed to the writing of the manuscript. ÁC, PM, and NG analyzed data. ÁC, NG, GM, FP, AP, AS, and PM contributed to the final approval of the version to be published.

## Funding

This work was supported by the Universidad de Buenos Aires [Grant number 20020170100562BA] and Agencia Nacional de Promoción Científica y Tecnológica [Grant number PICT 2016-0629 and PICT-2019-0139].

## Conflict of Interest

The authors declare that the research was conducted in the absence of any commercial or financial relationships that could be construed as a potential conflict of interest.

## Publisher’s Note

All claims expressed in this article are solely those of the authors and do not necessarily represent those of their affiliated organizations, or those of the publisher, the editors and the reviewers. Any product that may be evaluated in this article, or claim that may be made by its manufacturer, is not guaranteed or endorsed by the publisher.
